# Near-infrared optical absorption enhanced in black silicon via Ag nanoparticle-induced localized surface plasmon

**DOI:** 10.1186/1556-276X-9-519

**Published:** 2014-09-22

**Authors:** Peng Zhang, Shibin Li, Chunhua Liu, Xiongbang Wei, Zhiming Wu, Yadong Jiang, Zhi Chen

**Affiliations:** 1 State Key Laboratory of Electronic Thin Films and Integrated Devices, School of Optoelectronic Information, University of Electronic Science and Technology of China (UESTC), Chengdu, 610054, People's Republic of China; 2 Department of Electrical & Computer Engineering, University of Kentucky, Lexington, KY 40506, USA

**Keywords:** LSP, Ag nanoparticles, Black silicon, Chemical etching, Absorption

## Abstract

Due to the localized surface plasmon (LSP) effect induced by Ag nanoparticles inside black silicon, the optical absorption of black silicon is enhanced dramatically in near-infrared range (1,100 to 2,500 nm). The black silicon with Ag nanoparticles shows much higher absorption than black silicon fabricated by chemical etching or reactive ion etching over ultraviolet to near-infrared (UV-VIS-NIR, 250 to 2,500 nm). The maximum absorption even increased up to 93.6% in the NIR range (820 to 2,500 nm). The high absorption in NIR range makes LSP-enhanced black silicon a potential material used for NIR-sensitive optoelectronic device.

**PACS:**

78.67.Bf; 78.30.Fs; 78.40.-q; 42.70.Gi

## Background

The efficiency of the photovoltaic devices and photoelectronic detectors made from crystalline silicon (C-Si) decreases seriously due to its high reflectivity to the visible and near infrared light. In order to overcome the intrinsic disadvantage of silicon, many approaches have been explored. Most of these methods fall into two categories: 1) anti-reflection coating [1,2] and 2) light-trapping structure, like grating and period structures [3-6]. Among these methods, a simple and feasible way to obtain high absorption is to blacken the surface of crystalline silicon. In essence, black silicon is not a new material, but a surface modification microstructure of the Si material. Without any doping, the absorption of black silicon can be enhanced to over 90% in ultraviolet and visible range. There are different ways to fabricate black silicon, including reactive ion etching (RIE), electrochemical etching, acid etching, etc. [7-11]. Although it is easy to obtain black silicon by the methods mentioned above, the absorption in black silicon decreases sharply over 1,100 nm owing to the bandgap of silicon which is 1.12 eV. This high absorption is due to the micro/nanostructure on silicon, which blackened the surface, but not the change of bandgap. E. Mazur [12] proposed a new approach that sulfur was doped into silicon material by femtosecond laser pulses in SF_6_ environment, resulting in the high absorption in black silicon over the whole 250 to 2,500 nm wavelength. This high absorption in black silicon is ascribed to the impurity of bandgap levels induced by the doped chalcogenide [13,14]. However, the preparation of black silicon using a femtosecond laser is a high-cost and time-consuming process.

Recently, localized surface plasmon (LSP) also has gained wide attention ascribed to its enhancement in optical properties. The metallic nanostructures confine the charge density oscillations to produce the LSP effect. As the frequency of incident light matches the frequency of LSP, the light will be absorbed by the metal significantly. This phenomenon is called localized surface plasmon resonance (LSPR). LSPR improves the absorption capability through the enhancement of electromagnetic field near the nanoparticle or inside the nanoparticle. Meanwhile, the frequency of LSP is sensitive to the shape and size of metallic nanostructures as well as the dielectric constant of the surrounding material. LSP has been widely investigated due to the unique properties. The biosensors and solar cells based on metallic nanoparticles were reported since LSPR enhances the performance of such devices [15-20].

In this paper, we report a unique, efficient, and easy method to use LSP to enhance the absorption of black silicon in NIR. Due to the LSP effect, the black silicon with Ag nanoparticles indicates high absorption and low reflectivity in UV-Vis-NIR (250 to 2,500 nm) wavelength.

## Methods

The Ag thin film (10 nm) used in this work was deposited on silicon by thermal evaporation after the silicon substrate was cleaned by the RCA procedure. The post annealing was performed by rapid thermal processing at different temperatures (300°C, 400°C, 500°C, 700°C) for 3 min in nitrogen. All samples were etched in etchant acid solution (H_2_O_2_/H_2_O/HF/C_2_H_5_OH = 20:8:4:8 volume ratio; concentrations of H_2_O_2_ and HF are 30% and 40%, respectively) for 8 min at 35°C. A silicon sample without Ag nanoparticle on its surface was etched by an etchant containing HAuCl_4_ for 8 min at 35°C, and all samples were kept in ethanol. The surface morphology was characterized by field emission scanning electronic microscopy (SEM); the optical properties were measured using a UV-Vis-NIR spectrophotometer equipped with an integrating sphere detector.

## Results and discussion

**Figure 1:**
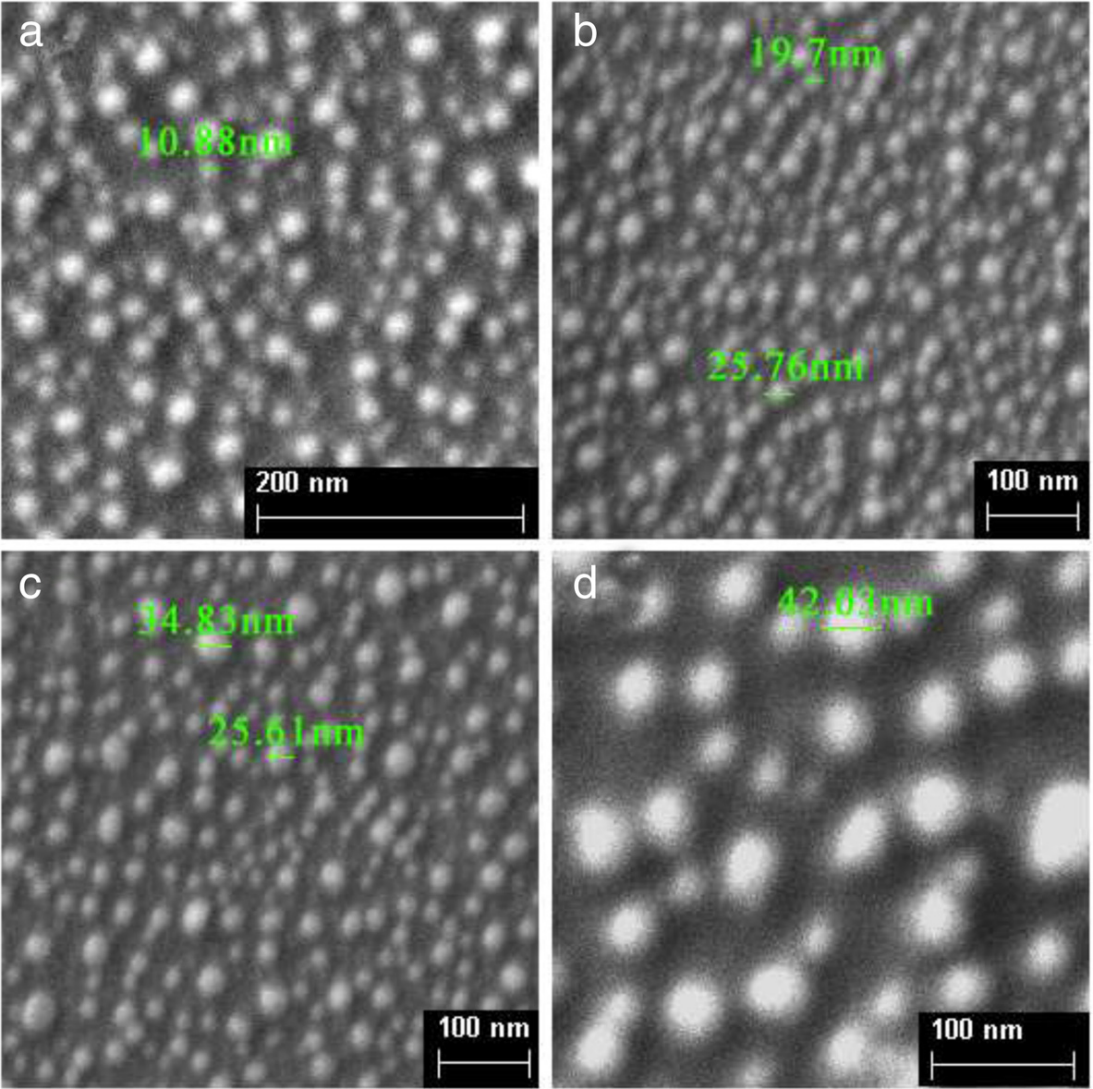
SEM images for samples annealed at different temperatures: (a) 300°C, (b) 400°C, (c) 500°C, (d) 700°C.

Figure 1 shows the Ag spherical nanoparticles on silicon surface produced by rapid thermal processing-induced stress. The Ag nanoparticles are much of a size under the same annealing temperature. The size of nanoparticles increases from 10.88 nm to 42.03 nm with the increase of annealing temperature, indicating higher annealing temperature produced larger particles. The space between two particles also depends on the annealing temperature. The result from the SEM images shows wider space between two nanoparticles as the annealing temperature increases.

The samples were etched by an etchant and contained HF and H_2_O_2_ after the annealing treatment. Lots of bubbles escaped from the surface once the samples were immersed in the etchant. During the etching process, the surface of the sample changed from a flat, light gray mirror finish, to a visibly matte black or dark gray surface. Meanwhile, the color of the etching solution changed from transparent to light purple.

**Figure 2:**
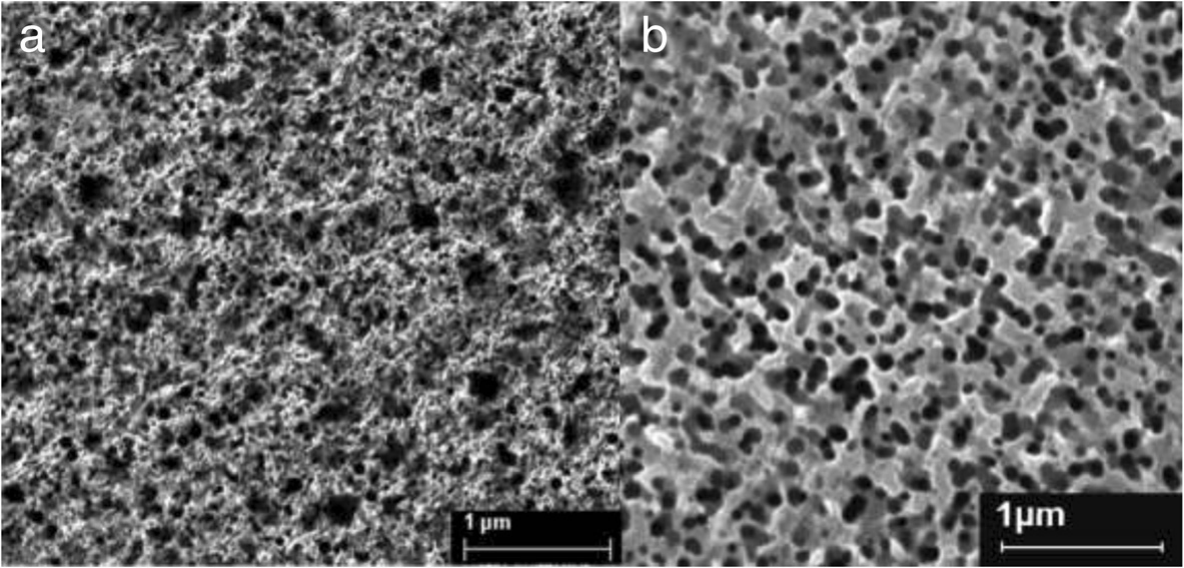
The SEM images of samples (a) 500°C and (b) silicon without Ag nanoparticles covered after chemical etching.

As shown in Figure 2, the nanopores formed on silicon film causes the black surface of silicon. The size of nanopores depends on the size of Ag nanoparticles. In the same annealing time, the size of nanoparticles produced from RTP process increases with the annealing temperature. The Ag nanoparticle forms the primary cell with Si in contact area when the substrates are immersed in the etchant, where the Ag nanoparticle acts as cathode to reduce H_2_O_2_. Si under the noble metal is oxidized by some charge transfer between Ag nanoparticles. The silicon oxides are dissolved by HF and nanopores are produced; more details can be found elsewhere [21,22]. Si covered by metal nanoparticles dissolves faster than the regions without nanoparticles according to the primary cell principle, resulting in nanopores on the surface [23]. The Ag nanoparticles sink into the bottom of the nanopores in black silicon.

**Figure 3:**
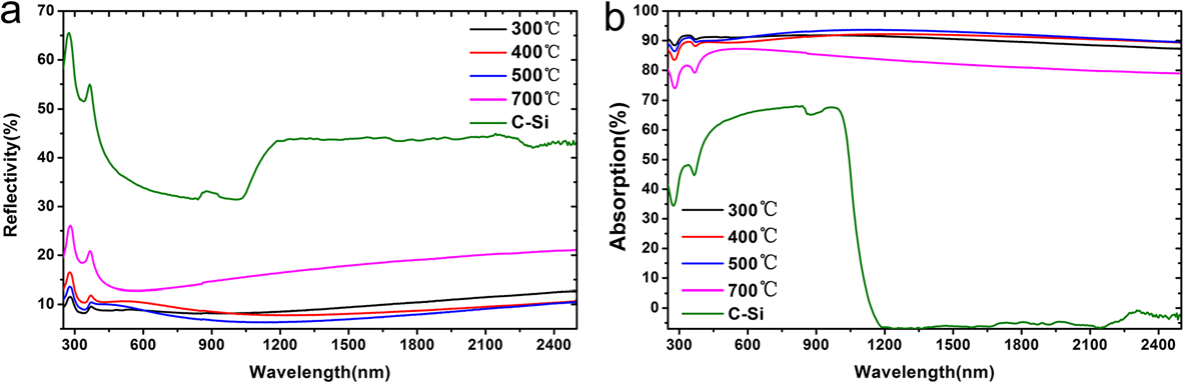
Reflectivity (a) and absorption curve (b) of samples annealed at different temperatures.

The optical properties of the samples are evaluated by measuring the infrared absorptance through a UV-Vis-NIR spectrophotometer equipped with an integrating sphere detector. The diffuse and specular reflectance (*R*) and transmittance (*T*) are measured with a 2 nm step length in the wavelength range of 200 to 2,500 nm to determine the absorptance (*A* = 1 - *R* - *T*). Figure 3 displays the reflectivity and absorption curves of samples annealed at different temperatures.

**Figure 4:**
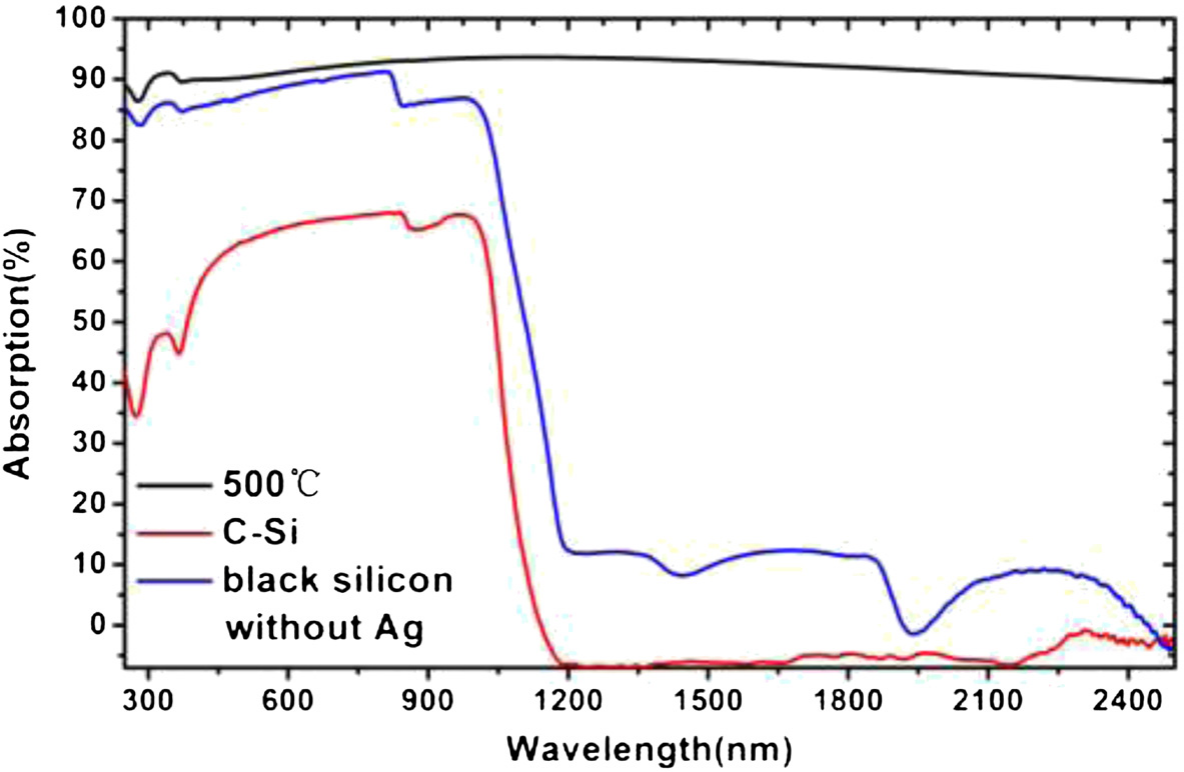
Absorption curve of sample 500°C, black silicon without Ag and C-Si.

As shown in Figure 3a, the reflection is obviously suppressed owing to the rough surface produced by Ag nanoparticle-induced chemical etching. The reflection of C-Si is much higher than that of black silicon. For the Ag nanoparticle-embedded black silicon, the size of nanoparticles differ with varying annealing temperature. The difference in reflectivity and absorption is ascribed to the different sizes of Ag nanoparticles and nanostructures. The sample annealed at 700°C indicates the highest reflectivity ascribed to the large-sized nanoparticles produced by the highest annealing temperature used in this work [24]. The average absorption of all Ag nanoparticle-induced black silicon is high, up to 91.8% in the range of 250 to 2,500 nm.Figure 4 shows the absorption curve of black silicon, Ag-embedded black silicon as well as crystalline silicon. The absorption range of Ag-embedded black silicon is obviously extended from UV to NIR wavelength. The additional absorption to wavelength over 1,100 nm can be attributed to the LSP in Ag nanoparticles.

After chemical etching, Ag particles are distributed in black silicon unevenly, and the particles can be approximately regarded as surrounded by silicon material. According to the MIE theory and modified Drude model, the frequency of surface plasmon resonance of a single isolate particle is described as follows:

(1) $$\omega_{\mathrm{sp}}^{2}=\frac{\omega_{p}^{2}}{1+\epsilon_{1}^{b}+2\epsilon_{m}}-{\left(\frac{1}{\tau_{0}}+\frac{V_{f}}{d}A\right)}^{2}≅\frac{\omega_{p}^{2}}{1+\epsilon_{1}^{b}+2\epsilon_{m}}-{\left(\frac{V_{f}}{L}+\frac{V_{f}}{d}A\right)}^{2}$$

where $\omega_{p}={\left(n_{e}e^{2}/\epsilon_{0}m\right)}^{\frac{1}{2}}$ is the free electron plasma frequency (for Ag, *ω*_*p*_ = 1.37036 × 10^16^ rad/s). *ϵ*_*m*_ is the dielectric function of Si. *ϵ*_1_ is the real part of the dielectric function of the Ag nanoparticles. *ϵ*^*b*^ represents the impact of electron interband transition on the dielectric function. *V*_*f*_ is the Fermi velocity (1.4 × 10^6^ m/s for Ag), *L* is the mean free path of the electron in bulk material, *d* is the radius of nanoparticles [25-27]. For most metals at room temperature, the plasma frequency is usually in the visible and UV regions, approximately from 3 to 20 eV. For Ag particles (5 nm < *d* <50 nm) in vacuum, the frequency of plasmon resonance approximately corresponds to 350 nm [28,29]. When *ϵ*_*m*_ or refract index $\left(\epsilon_{m}=n_{m}^{2}\right)$ increases, the frequency of plasmon resonance could be shifted to NIR [30,31].

Furthermore, as shown in Figure 1, particles are close to each other, the interparticle distance is less than a radius of particles, and this situation remains roughly the same after etching [21], which means interaction between particles cannot be ignored [32]. Considering the interaction between particles, the resonance frequency of particle *i* can be explained as follows:

(2) $$\omega_{i}=\sqrt{\omega_{\mathrm{sp},i}^{2}-\frac{\omega_{p}}{4\pi \epsilon_{m}}\sum_{i\neq j}\frac{V_{j}\left(3\text{cos}\theta_{i,j}-1\right)}{d_{i,j}^{3}}}$$

where *ω*_sp,*i*_ is the resonance frequency of particle *i* without considering interparticle interaction, *θ* is the angle between the incident polarization and the axis connecting two particles. *V* represents the volume of the particle. The second part of the equation describes the interaction between particles. This clearly shows that interaction between particles could reduce the resonance frequency and result in the additional red shift of the plasmon resonance frequency [33].

As a result, frequency of plasmon resonance in Ag nanoparticles shifts to NIR, mainly because of the interaction between particle size, high refractive index surrounding the material, and interparticle coupling. Besides, the refractive index of black silicon is the gradient [8] and the Ag nanoparticles are unevenly distributed, which means the plasmon-enhanced absorption peak in NIR can be the superposition of many different peaks. As the incident wavelength increases, the decay rate of the free electrons of the dielectric constant of the silver increases, shortening the life of the plasma and broadening the plasma resonance band [34]. These peaks would overlap with each other. Therefore, the high absorption (over 90%) in NIR range presented in this work is ascribed to the enhancement of LSP induced by nanoparticles embedded into the surface of black silicon. It is different from the high absorption induced by the impurity of the bandgap level in black silicon attributed to chalcogenide doping by the femtosecond laser process [12,13,35-37].

## Conclusion

In this paper, instead of chalcogenide-doped black silicon prepared using a femtosecond laser, we report a black silicon with Ag nanoparticles embedded showing high absorption from UV to NIR (250 to 2,500 nm) owing to the enhancement induced by LSP. The LSP-enhanced absorption is high, up to 93.6%, and the average is 91.8% over the 250 to 2,500 nm. The LSP-enhanced black silicon provides an alternative approach to producing high absorption in NIR range through a simple and cheap method.

## Competing interests

The authors declare that they have no competing interests.

## Authors’ contributions

PZ carried out the experiments and drafted the manuscript. SL and CL participated in the design of the experiments and revised the manuscript. XW, ZW, YJ, and ZC participated in the experimental design and coordination. All authors read and approved the final manuscript.
